# CK and LRRK2 Involvement in Neurodegenerative Diseases

**DOI:** 10.3390/ijms252111661

**Published:** 2024-10-30

**Authors:** Valentina Bova, Deborah Mannino, Anna Paola Capra, Marika Lanza, Nicoletta Palermo, Alessia Filippone, Emanuela Esposito

**Affiliations:** 1 Department of Chemical, Biological, Pharmaceuticals and Environmental Sciences, University of Messina, Viale Stagno d’Alcontres, 98166 Messina, Italy; valentina.bova@unime.it (V.B.); deborah.mannino@unime.it (D.M.); annapaola.capra@unime.it (A.P.C.); mlanza@unime.it (M.L.); eesposito@unime.it (E.E.); 2 Department of Biochemical, Dental, Morphological and Functional Imaging, University of Messina, Via Consolare Valeria, 98125 Messina, Italy; nicoletta.palermo1@unime.it

**Keywords:** casein kinases, leucine riche-repeat kinase, neurodegeneration, degradative pathways

## Abstract

Neurodegenerative diseases (NDDs) are currently the most widespread neuronal pathologies in the world. Among these, the most widespread are Alzheimer’s disease (AD), dementia, Parkinson’s disease (PD), amyotrophic lateral sclerosis (ALS), and Huntington’s disease (HD)—all characterized by a progressive loss of neurons in specific regions of the brain leading to varied clinical symptoms. At the basis of neurodegenerative diseases, an emerging role is played by genetic mutations in the leucine-rich repeat kinase 2 (LRRK2) gene that cause increased LRRK2 activity with consequent alteration of neuronal autophagy pathways. LRRK2 kinase activity requires GTPase activity which functions independently of kinase activity and is required for neurotoxicity and to potentiate neuronal death. Important in the neurodegeneration process is the upregulation of casein kinase (CK), which causes the alteration of the AMPK pathway by enhancing the phosphorylation of α-synuclein and huntingtin proteins, known to be involved in PD and HD, and increasing the accumulation of the amyloid-β protein (Aβ) for AD. Recent research has identified CK of the kinases upstream of LRRK2 as a regulator of the stability of the LRRK2 protein. Based on this evidence, this review aims to understand the direct involvement of individual kinases in NDDs and how their crosstalk may impact the pathogenesis and early onset of neurodegenerative diseases.

## 1. Introduction

The central nervous system (CNS) is the control center of the entire human body and consists of the brain and spinal cord. The CNS is particularly susceptible to neurodegenerative diseases (NDDs), which today represent pathological conditions mostly widespread in industrialized countries, affecting more than a billion people in the world [1,2]. Although the literature reports that aging is the main risk factor of NDDs, there are some studies that have reported comorbidity events in elderly patients [3,4]. An example was provided by José A. Santiago [5], in which some metabolic diseases (such as type II diabetes), gastrointestinal diseases, states of depression and anxiety, but also chronic inflammatory processes, can be associated with a high risk of developing Alzheimer’s disease (AD). NDDs are characterized by a progressive loss of neuronal structure and function. Given the complex nature of the CNS, research is constantly evolving to provide high specificity in the treatment of NDDs [6,7,8]. At the basis of NDDs, an emerging role is given to genetic mutations, in particular the mutation of the gene that codes for α-synuclein (α-syn) [9], the microtubule-associated protein Tau (MAPT), and leucine-rich repeat kinase 2 (LRRK2) [9].

Mutations in the *LRRK2* gene represent genetic risk factors for the development of neurodegenerative diseases, particularly in Parkinson’s disease (PD) [10]. Structurally, LRRK2 is made up of a set of guanosine triphosphatases (GTPases), kinases, and scaffold domains [11] and it has been made clear that the possible pathological function of LRRK2 is closely related to the hyperactivity of the kinase [12]. This results in neuronal cell death, impaired dopamine neurotransmission, and defects in protein synthesis, degradation, inflammatory responses, and oxidative damage [10,13].

Additionally, casein kinases (CK), a family of proteins that have also been implicated with emerging roles in neurodegeneration at the CNS level—casein kinase1 (CK1) and casein kinase 2 (CK2), are the main proteins of this family and are involved at the forefront of NDDs [14]. CK1 and CK2 modulate various biological processes, including protein phosphorylation through the modification of different residues including Ser, Thr, and Tyr [15,16]. Considering their fundamental role, recent studies have highlighted the importance of CK1 and CK2 in the development of neurodegenerative diseases [14]—and regarding CK1 particularly, several studies state that of the four isoforms (CK1α, CK1β, CK1δ, and CK1ε), the most involved in the development of various neurodegenerative pathologies are CK1δ and CK1ε, but with CK2 having isoforms CK2α, CK2α’, CK2β, that are involved at the forefront.

A crosstalk was demonstrated between the CK protein and LRRK2—knowing that *LRRK2* is a gene relevant to PD, it is itself phosphorylated and modulated in several signaling pathways [17]. Although more in-depth studies are needed to better clarify which kinase is responsible for controlling LRRK2 signaling, it has been well established that CK1, especially the CK1α protein, is the kinase primarily responsible for LRRK2 phosphorylation in S910 and S935. A recent study concluded that there is a positive correlation between CK1a and LRRK2 phosphorylation [18]. Therefore, understanding the role of LRRK2 and CK1 and their crosstalk in neurodegenerative diseases could be important for the development of new therapeutic approaches. In this review, we would specifically highlight the involvement of LRRK2, CK1, and CK2 in NDDs by understanding their roles independently or in dual action.

## 2. Neurodegenerative Pathologies: Points on Molecular Pathways and Players

### 2.1. Role of Autophagy and Apoptosis in Neurodegeneration

**Figure 1:**
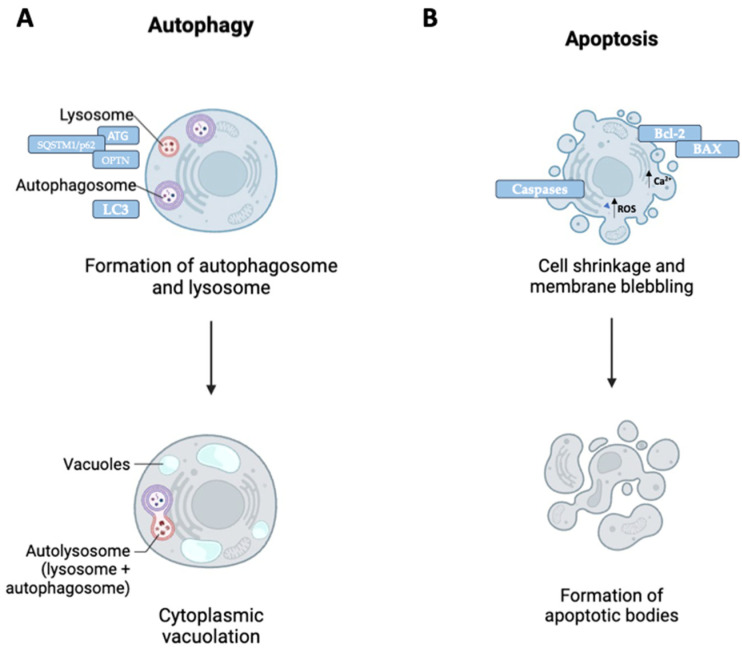
Autophagy and apoptosis diagrams. (**A**) Misfolded protein aggregates are tagged with ubiquitin chains, recognized by receptors such as SQSTM1/p62 and optineurin (OPTN). These receptors link ubiquitinated cargo to autophagosomes by interacting with the autophagosome-associated protein LC3, facilitating selective degradation. (**B**) The process of apoptosis is initiated by signals involving proteins of the Bcl-2 family. Proapoptotic proteins, such as Bax, promote the permeabilization of the outer mitochondrial membrane. This leads to the activation of proapoptotic molecules, such as caspases, which determine the controlled degradation of damaged cells.

Many neurodegenerative diseases are caused by defects in cellular functions linked to altered protein homeostasis and the consequent progressive accumulation of pathological intracellular materials. In this context, autophagy and apoptosis play a key role in the control of various physiological processes of cellular homeostasis [19,20]. Numerous pieces of evidence have shown that in neurodegenerative disorders such as AD [21], PD [22], HD [23], ALS [24], spinal muscular atrophy (SMA) [25], and diabetic neurological disorder, autophagy and apoptosis pathways are altered. Autophagy is a vital intracytoplasmic protein degradation pathway through which cellular contents are recycled and delivered from double-membrane vesicles, called autophagosomes, to the lysosome organelle for degradation by lysosomal enzymes [26,27]. This process is regulated by a series of proteins defined as autophagy-related proteins (ATGs) [28]. Similarly, apoptosis is also essential for the defense and maintenance of healthy neuronal cells as it is responsible for the elimination of damaged cells. The apoptosis pathway is initiated by a series of signals, as well as Bcl-2 proteins, high levels of Ca^2+^, and reactive oxygen species (ROS), which leads to the discharge of many prodeath molecules in the cytoplasm [29] (Figure 1).

It is now known that autophagy plays a key role in the removal of aggregation-prone proteins, and the breakdown of this pathway is involved in progressive and early-onset neurodegeneration in the brain. In this context, mutations in the genes encoding phosphatase and putative tensin homolog-induced kinase 1 (PINK1) and the Parkin E3 ubiquitin ligase (Parkin) are associated with autosomal recessive forms of early-onset PD [30]. Numerous studies have shown that LRRK2 kinase mutations are the main cause of both sporadic and familial forms of PD [31,32,33,34]. In particular, G2019S mutation of LRRK2 causes an increase in the kinase activity of this protein. The increase in kinase activity of LRRK2 has been proposed as responsible for its neurotoxic effect. It has been shown that increased kinase activity of LRRK2 blocks the formation of the Chaperone-mediated autophagy (CMA) translocation complex in the lysosomal membrane. This results in the reduced functionality of CMA. Therefore, damaged proteins or misfolded proteins (such as α-synuclein) are no longer degraded properly and accumulate, resulting in the formation of Lewy bodies. Thus, it can be suggested that mutations in LRRK2 may play a role in influencing the formation of Lewy bodies in PD [35,36].

Moreover, it has been observed that the dysregulation of CK1 and CK2 can contribute to the progression of these diseases through inappropriate cell survival or inability to eliminate tau protein aggregates. Therefore, CK1 and CK2 dysregulation may alter the phosphorylation of autophagic proteins, inhibiting the correct initiation of autophagy [37]. Since the activity of CK1 and CK2 influences the activity of LRRK2 and vice versa, it would not be surprising that they act on common substrates [18,38,39]. De Wit, T. et al. [39] and Ruth Chia et al. [18] identified CK1, an upstream kinase of LRRK2, capable of regulating the stability of the LRRK2 protein in vitro and in vivo. The effects of pharmacological inhibition of CK1 on the LRRK2 kinase were also evaluated, showing that the inhibition of CK1 could cause a rapid and marked reduction in LRRK2 protein levels. Similarly, inhibition of the LRRK2 kinase triggers a cascade that leads to reduced CK1-mediated phosphorylation. Furthermore, the study demonstrated that several pathogenic LRRK2 mutants are not destabilized by inhibition of this kinase activity; however, they are highly sensitive to CK1 inhibition. Therefore, it is hypothesized that pharmacological inhibition of LRRK2 kinase induces N-terminal changes that lead to reduced interaction with CK1, resulting in reduced heterologous phosphorylation and, ultimately, degradation of the LRRK2 protein. This is consistent with the data obtained by Ruth Chia et al., where the inhibition of LRRK2 kinase leads to reduced LRRK2-CK1 interaction. In particular, the study by Ruth Chia et al. states that CK1α is an upstream kinase responsible for phosphorylation of LRRK2 at several constitutive phosphorylation sites. Although it is not yet clear which specific phosphorylation sites are involved in this process, they are thought to be located outside the N-terminal region of LRRK2.

The same argument can also be applied to the autophagy process, where CK2 modulates the activity of autophagic proteins, consequently influencing the activity that LRRK2 has on mitophagy. The combined action of these kinases could influence the removal of toxic protein aggregates in these diseases.

### 2.2. Contribution of AMPK Pathway in Neurodegeneration

The 5′-adenosine monophosphate-activated protein kinase (AMPK) is an important gatekeeper of cellular energy status [40], consisting of a catalytic subunit α and two regulatory subunits β and γ [41]. It is expressed in almost all immune cells and regulates the inflammatory response through the regulation of several pathways, in part by inhibiting, downstream, the nuclear factor κB (NF-κB) pathway, as well as acting as a negative regulator of toll-like receptors (TLRs) [42]. In physiological conditions, AMPK directs various biological processes, of which the main ones are lipid metabolism, inhibiting anabolism by promoting lipid catabolism [43] and stimulation of glucose absorption in skeletal muscles (Figure 1) [44]. Furthermore, AMPK reduces protein synthesis during the transcription phase [42] and activates autophagy through direct and indirect activation of ULK1, a mammalian homolog of ATG1 [45,46]. It has been reported that it stimulates the processes of apoptosis and autophagy through the inhibition of the mechanistic target of rapamycin (mTOR), which normally regulates cellular metabolic homeostasis, insulin secretion, insulin resistance, autophagy, and apoptosis [47]. At the CNS level, studies report that AMPK has a protective effect against neuroinflammation [48]. This action is due to the presence of its a and b subunits in hippocampal neurons [49], where, as previously mentioned, AMPK acts as a regulator of cellular homeostasis. Furthermore, Culmsee, C. et al. demonstrated that AMPK protects neurons from different metabolic and excitotoxic insults (pathogenesis of neurodegenerative diseases) by suppressing the levels of α1 and α2 subunits using antisense technology [49].

#### 2.2.1. CK1/2 and LRRK2 Influence AMPK Activity

From a molecular point of view, it was found that CK1 and CK2 isoforms influence AMPK activation, phosphorylating the AMPK protein at Ser/Thr residues, affecting its stability and activity, which in turn can be inhibited or activated, depending on the context in which they are found [50]. Consequently, activated AMPK can phosphorylate target substrates of CK1 and CK2, thereby influencing the pathways into which these kinases converge [51]. Another important molecular factor that influences AMPK-controlled processes is hyperactive LRRK2, which, in the context of PD, would impair the AMPK-mediated autophagic response [52] causing cellular stress and, thus, accelerating neurodegeneration. Furthermore, LRRK2 affects the ability of AMPK to control normal vesicular trafficking and mitochondrial activity [53].

#### 2.2.2. The Effect of Crosstalk Between LRRK2 and CK1/2 on AMPK

The crosstalk between AMPK, LRRK2, and CK1/CK2, in this context, is complex and significant, and plays a fundamental role in biological processes such as energy homeostasis, apoptosis, and autophagy. The crosstalk LRRK2 and CK1 and CK2 can have significant effects on AMPK signaling. In this regard, phosphorylation of LRRK2 on CK1 and CK2 is able to influence mitochondrial function and cellular energy balance controlled by AMPK. This is similar for autophagic proteins (beclin-1 or ULK1) [54], whose activity modulated by AMPK is upstream influenced by the interaction of LRRK2 with CK1 and CK2. Therefore, the interaction between these kinases is of considerable importance, especially in the context of neurodegenerative diseases, because modulating the balance between them may offer a potential therapeutic strategy. Despite promising preclinical data, sometimes also supported by clinical studies, further research is needed to elucidate the precise mechanisms underlying AMPK-mediated neuroprotection in neurodegenerative diseases and to optimize therapeutic interventions targeting AMPK signaling.

#### 2.2.3. AMPK and Its Involvement in Neurodegenerative Diseases

There are several other factors capable of activating AMPK. These factors include overnutrition, obesity, calorie restriction, and inflammation [55]. In this regard, there are conflicting reports on the benefits or harmful effects of AMPK in distinct pathological conditions [42]. As evidence, a study conducted on patients with type 2 diabetes found an association between inflammatory genes and a reduction in AMPK activity due to high oxidative stress in the adipose tissue of these patients [56,57]. Proinflammatory cytokines, such as IL-6 and TNF-a, activate IkB kinase, which phosphorylates IkB-a, releasing NF-κB, consequently translocating into the nucleus, promoting the inflammatory process [58]. Several studies have shown that AMPK indirectly inhibits NF-κB activation through downstream pathways that suppress the expression of inflammatory genes [58]. In support of this finding, Entezari M. et al. [59] showed that the administration of Metformin, an antidiabetic drug par excellence, induces AMPK signaling, thereby reducing serum IL-6 and TNF-a levels; in addition, the use of Simvastatin by inducing AMPK signaling exerts an anti-inflammatory effect, where it has been shown to reduce NF-κB levels [60]. In addition, there is a correlation between high glucose concentrations and the development of neurodegenerative diseases, such as dementia and Alzheimer’s [61], thus suggesting that nervous system degeneration may be the result of impaired cellular metabolism [42], as occurs in diabetes. Clinical studies have provided insights into the role of AMPK in the context of AD, reporting a close correlation between AMPK regulatory dysfunction and the development of the pathology, understood as neuronal dysfunction, synaptic deterioration, mitochondrial dysfunction, oxidative stress, and abnormal protein aggregation [41]. This study, therefore, reports the protective role of AMPK by attenuating the neuroinflammation that characterizes AD, phosphorylation of the tau protein, and metabolism of the Ab protein [41]. In the context of PD, where an alteration of cellular homeostasis is observed, studies have reported that an overactivation of AMPK leads to an accumulation of α-synuclein aggregation [62]. Although the role of AMPK in PD is known, to date there have been no clinical studies supporting this finding. Furthermore, in confirmation of what has just been mentioned, in the mouse model of PD, MPTP-induced neurodegeneration stimulated the production of AMPK, thus contributing to neuronal damage, although AMPK overexpression in SHSY5Y neuronal cells played a neuroprotective role [63]. This has led to the likely conclusion that the upregulation of AMPK in damaged neurons is a compensatory protective effect, used as a strategy to increase energy supply and neuronal survival [63]. To date, it is not entirely clear whether AMPK at the neuronal level plays a protective or harmful role [64,65].

Mitochondrial dysfunction and oxidative stress are processes that neurodegenerative diseases have in common. In fact, in this regard, another neurodegenerative disease in which there is clear evidence of the involvement of AMPK is amyotrophic lateral sclerosis (ALS) [66]. In response to these dysfunctional processes, there are the production of reactive oxygen species (ROS) and energy deficit. Similarly to the other neurodegenerative diseases mentioned above, it has also been shown for ALS that AMPK activation restores mitochondrial activity, reducing oxidative stress in ALS models [66,67]. Active AMPK stimulates the autophagic process, facilitating the removal of protein aggregates and the promotion of neuronal survival in ALS models. Furthermore, it has been demonstrated that AMPK can attenuate neuroinflammation and consequent neuronal death, thanks to its ability to act on the inflammatory pathway and inhibit neuronal apoptosis [68].

## 3. CK Superfamily as Targets for Neurodegenerative Disorders Protein Kinases CK1 and CK2 and CNS Disorders

Although it is not clear what the specific mechanism of α-syn phosphorylation is, scientific evidence reports that in PD, the phosphorylation of α-syn is regulated by important protein kinases [69]. In particular, the main protein kinases that take part in this event are represented by proteins belonging to the polo-like kinase (PLK), casein kinase (CK), and G protein-coupled receptor kinase (GRK) families [70]. Additionally, extracellular signal-related protein kinase (ERK), c-Jun N-terminal kinase (JNK), p38, protein kinase B (PKB), and mammalian target of rapamycin (mTOR) [69,71] regulate the processes of inflammation, autophagy, and apoptosis in PD. In this review, we will address the role of CKs in CNS disorders in more detail. The CK family consists of Ser/Thr enzymes expressed ubiquitously and involved in diverse biological processes that occur in most eukaryotic cells [72]. The members constituting this family are represented by CK1 and CK2, whose differences concern structure, localization, and function [73].

### 3.1. CK1 Family

**Figure 2:**
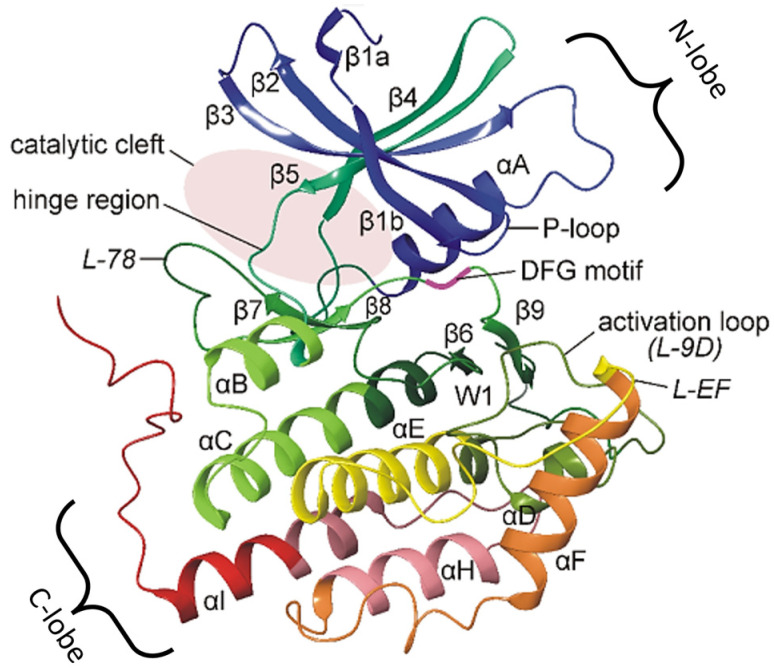
Structure of CK1δ. The structure is composed of two main domains: a small N-lobe and a large C-lobe. The N-lobe is composed mainly of β-sheets, while the C-lobe is composed predominantly of α-helices. These two lobes are essential for the formation of the catalytic cleft, which is the active site where CK1δ binds to ATP and its target substrates. This cleft facilitates kinase activity by allowing the transfer of a phosphate group from ATP to the substrate, thereby regulating various cellular processes [77].

In mammals (membrane, nucleus, cytoplasm, mitotic spindle), seven isoforms have been identified, encoded by different genes: alpha, beta 1, gamma 1, gamma 2, gamma 3, delta, and epsilon, which differ in their molecular weight [74,75]. Structurally, CK1 consists of a small N-terminal lobe, a large C-terminal lobe, and a catalytic cleft where ATP and substrates bind (Figure 2) [76].

Although a comparison between the enzymatic kinetics of each isoform is lacking, they all possess a high degree of homology in their kinase domains, with a strong tendency to bind “primed” and pre-phosphorylated substrates. Despite this binding preference, they are also capable of phosphorylating related unprimed sites under certain conditions [76]. CK1 family members catalyze phosphate transfer onto the Ser/Thr residues of their protein substrates using ATP exclusively as a phosphate donor source [78]—since most cellular proteins feature at least one of the CK1 consensus motifs, hundreds of CK1 substrates have been reported [72]. Precisely for this reason, the lack of isoform-selective CK1 inhibitors does not allow us to fully understand which CK1 isoform is exactly the physiological kinase for each of the identified substrates [79]; however, in recent years, scientific evolution has led to the generation of specific inhibitors of the CK1 isoform [77]. To understand the substrate specificity of the CK1 isoforms, the first attempts concerned the priming phosphorylation at position-3 of the CK1 phosphorylation site, in which the consensus sequence pS/pT-XXS*/T* is present (X = any amino acid and S*/T* = CK1 phosphorylation residues), and this represents an optimal motif of CK1 phosphorylation [80]. It was subsequently discovered that a group of N-terminal acidic residues at position-3 can replace the priming phosphorylation event, compared to the Ser/Thr phosphorylation target sites [81]. Following this discovery, studies on substrate specificity continued, leading to the common conclusion that all CK1 isoforms phosphorylate Ser/Thr residues that are not defined by a specific sequence motif. Therefore, what has just been reported helps us understand how the phosphorylation of specific substrates in cells is under the control of other factors, such as the determinants of their subcellular distribution and substrate recruitment [82]. Despite the similarity between the different CK1 isoforms, there are inconsistencies between in vitro and in vivo studies in this regard [79,83] due to intracellular regulatory mechanisms involved in the modulation of CK1 isoforms. Since the kinase domain of CK1 isoforms represents the majority of the protein sequence, regulatory domains within the protein sequence, which are prevalent in many other kinases, are smaller in size in CK1 isoforms. Not only that, the noncatalytic C-termini of the CK1 isoforms are very varied and are not conserved domains between isoforms. This would suggest that the C-termini are involved in the regulation of substrate recognition and modulation of kinase activity for some CK1 isoforms [84]. There is a way to modulate CK1 function in cells and it involves the use of scaffolding or anchoring proteins, which ensure the correct positioning of the protein complexes [85]. Among these, some scaffolds can act as signalosome assembly structures, directing functional enzymes toward their substrate proteins in the same way as kinases, thus increasing the efficiency of the reaction through spatial control [86]. Thus, scaffolds regulate different biological processes of CK1 isoforms, such as acting as a bridge or barrier between isoforms and their substrates or are capable of binding and sequestering CK1 isoforms at discrete subcellular locations [87].

### 3.2. CK2 Family

**Figure 3:**
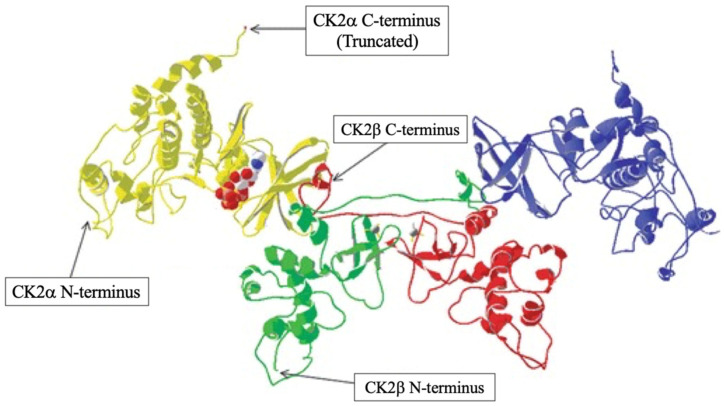
Structure of CK2. The structure is composed of two catalytic subunits α/α’ shown in blue and yellow and and two regulatory β subunits shown in green and red. The catalytic α/α’ subunits have an N lobe made up of β-sheets and a C lobe containing α-helices. Between these two lobes is the catalytic cleft where ATP binds to facilitate phosphorylation. The regulatory β subunits have no kinase activity but play a key role in stabilizing the enzyme [95].

Unlike CK1, CK2 is a tetrameric enzyme made up of two catalytic subunits (CK2α and CK2α′), which have 90% sequence homology in the N-terminal region, and a regulatory subunit (CK2β dimer), completely different from another two [88,89] (Figure 3). Like CK1, CK2 is also present in many organisms, tissues, and almost all subcellular compartments. Furthermore, CK2 phosphorylates more than 300 substrate proteins involved in diverse cellular processes such as cell division, proliferation, apoptosis, and DNA repair [90]. Regarding the β subunit, its regulatory functions are limited to preserving the stability of the enzyme and guiding the selection of substrates, which is why CK2 is constitutively active and catalytically competent even in its monomeric form [90]. The α subunits do not require the functioning of the β regulatory subunits, which allows the dimers to form catalytic domains independent of β subunit transcription. Indeed, CK2 is constitutively active and catalytically competent even in the monomeric form [91]. Although it is not yet entirely clear, there is a functional difference between the two α subunits; an example is given by Caspase3, which is phosphorylated by α’-based tetramers compared to α-based tetramers [92]. Like CK1, CK2 also uses ATP or GTP as a source of phosphate for its function. Beyond this, CK2 is also involved in the regulation of cell growth, proliferation, and suppression of apoptosis [93]. With regard to signal transduction, being constitutively active, CK2 does not respond to stimuli coming from outside the cell but is already ready to carry out its action on otherwise activated pathways, further confirming its ability to control various biological processes [94].

### 3.3. Participation of Both CK1 and CK2 in Neurodegenerative Diseases

The CK family is involved not only in a variety of signaling pathways but also in inflammation, cancer, and neurological diseases. In particular, CK1 and CK2 play an important role in the development of numerous neurodegenerative diseases, such as AD, PD, HD, and ALS, characterized by a loss of neurons in particular regions of the CNS [14]. Several studies have been carried out that identify different substrates that play a crucial role in the function and activity of the neuronal and synaptic network [14]. In addition to neuronal death, the abovementioned diseases also have characteristics in common, such as deficits in neurotransmitter systems, protein misfolding, and aggregation [96]. Based on the brain areas involved, the clinical symptoms of each of these diseases differ; AD is characterized by cognitive deficit, linked to memory loss, especially in the short term; the main symptoms of PD are linked to debilitating tremors, while ALS is characterized by a muscular deficit [73].

### 3.4. CKs in PD

In the CNS, glutamate is the main excitatory neurotransmitter, which directs the correct activity of neurons, especially at the striatum level [73]. In particular, studies conducted on human brain tissue report that it is the phosphorylation of α-syn Ser129 by CK1 and CK2 that precedes the accumulation of Lewy bodies (LBs) at the nigrostriatal level, thus favoring the progression of the disease [70,97]. Studies in the literature report that all CK1 isoforms are expressed in the striatum and also in the cortex regulating glutamatergic synaptic transmission, mediated by N-methyl-D-aspartate (NMDA) receptors [73]. In this regard, given the affinity of the interaction between the NMDA receptor and CK1, one might think that CK1 can control normal physiological processes related to movement by binding to the NMDA receptor [98]. Another factor that could suggest the involvement of CK1 in PD is the genetic component, in particular the mutation of the *Parkin* gene, whose mutation causes juvenile-onset autosomal recessive Parkinsonism [99,100]. It was demonstrated by Daniel I. Perez et al. that the parkin protein normally has a neuroprotective function, although its real function remains unclear. Its involvement in PD is due to point mutations that reduce its solubility promoting its aggregation. CK1 phosphorylating the Parkin protein in Ser/Thr sites decreases its solubility, leading to its aggregation and inactivation. Furthermore, CK1 dysregulation could amplify cellular stress and consequent DNA damage, increasing the likelihood of mutations in the *Parkin* gene [83]. Nowadays, there are numerous approaches to genome editing in PD. Among these, the most relevant and the one that has allowed us to open the way to new therapeutic approaches is the use of the CRISPR/Cas9 technique [101]. Through this technique, it is possible to modulate the expression of specific genes (upregulation and/or downregulation). In the case of PD, knockout models have been created through this technique to better understand, for example, mitochondrial dysfunction [102], thus helping researchers to identify new potential therapeutic targets. Another knockout model created with the CRISP/Cas9 technique involves the *SNCA* gene [103], encoding for α-syn, in order to study its aggregation and involvement in PD. Therefore, genome editing techniques and their combination with therapeutic approaches allow the development of targeted and personalized treatments for each individual patient while reducing the risk of developing side effects (Table 1) [102].

### 3.5. CKs in AD

Lately, research has focused on the involvement of CK1 and CK2 in tauopathies, such as AD. Like PD, AD is also a progressive neurodegenerative disease that affects neurons located in the hippocampus, neocortex, and other regions of the brain [104]. To date, the cause of AD is not entirely clear; what is known in the scientific field is that the development of AD is related to the accumulation of hyperphosphorylated Tau protein [105], which is polymerized into filaments and Ab plaques at the level of the hippocampus, compromising neuronal functionality [106]. There is clear evidence confirming the involvement of CKs in AD. Although CK1 and CK2 belong to the same family, they play different roles in this pathology [107], as CK1 is more involved in circadian disruption [108] and Tau protein phosphorylation [109], while CK2 plays a role in the development and progression of both the abnormal tau formation and the accumulation of amyloid-β plaques [89,110], neuroinflammation, and also cell survival [111], including the regulation of gene associated with the immune response, and is one of the factors responsible for neuroplasticity that ensures cell survival. All of these could influence the mechanisms that contribute to the development of AD. In this context, the brain regions most involved include the hippocampus [107] and the frontal cortex [112], which show alterations in CK2 concentration. To better understand the involvement of CK2 in AD, some immunoreactivity studies were conducted on brain tissue from Alzheimer’s patients [113]. The study shows how the concentration of CK2 decreased in AD, further confirming how Alzheimer’s can affect the activity of this protein. As mentioned above, although CK1 and CK2 have two independent pathways in AD, their diversity (both in terms of concentration levels in brain regions and mechanism of action) is not related to the development of the pathology. Moreover, studies have been conducted on the three isoforms of CK1 (CK1α, CKδ, and CDε) in Alzheimer’s patients, which were found to be upregulated 30-fold at the hippocampus level [83]. Furthermore, the CKδ isoform appears to be responsible for the phosphorylation of the Tau protein, resulting in the formation of neurofibrillary tangles [114], which is why CK1 has also been localized in bodies of granulovacuolar degeneration. Regarding the CK1ɛ isoform, this regulates the processing of the amyloid precursor protein (APP) [115]. Therefore, increased activity of CK1δ/ε isoforms leads to an accumulation of neuronal dopaminergic phosphoprotein (DARPP-32) phosphorylated by Thr-34. A further in vitro study reported the direct allosteric activation of CK2 by the Aβ peptide. CK2 directly phosphorylates Ser7 and Ser9 of presenilin-2 (PS-2), a protein that is part of the γ-secretase complex and involved in the processing of the Amyloid Precursor Protein (APP) [116].

The AD mouse model reported that CK2 directs the activity of α-secretase, which produces soluble APPα, by binding to the 5-hydroxytryptamine 4 (5-HT4) receptor, and this would lead one to think that improving CK2 activity would consequently reduce the formation of amyloid plaques. This finding would agree with the data obtained from the clinical study previously described. Nevertheless, opinions regarding the role of CK2 in AD are conflicting, as other studies suggest that CK2 inhibition has positive consequences on AD [104]. This latter thought is supported by a study in an in vivo model using a Protein phosphatase 2 (PP2A) inhibitor, SET. Specifically, it was shown that when CK2 phosphorylates Ser9 of SET, SET translocates into the cytoplasm where it binds and inhibits PP2A at the same time, causing hyperphosphorylation of the Tau protein. Mice, following PP2A inhibition, developed cognitive impairments characteristic of AD [104]. A study conducted by Sundaram et al. [105] reported that the inhibition of CK1δ/ε isoforms with their selective inhibitor, PF670462, improved cognitive function by reducing Ab plaques in a mouse model of AD [105]. Another important factor, believed to be responsible for the development of AD, is the mutation of the binding re-regulatory partners of CK1, such as a family with sequence similarity 83 member G (FAM83G), in which the binding is interrupted between the same and the CKα isoform. More in-depth genome editing studies are needed to better understand how dysfunctional CK1 signaling is closely related to AD development [72]. Therefore, given the relevant role of CK1 and CK2 in biological processes, only recently has research focused on developing inhibitors that are selective for each isoform, useful for blocking the progressive accumulation of neurofibrils in AD (Table 2).

## 4. Solid Links Between LRRK2 and PD

LRRK2 is a multidomain enzymatic protein with multifunctional kinase activity. Structurally, it is characterized by leucine-rich repeats (LRRs), a Roc GTPase domain, a COR domain (C-terminal of Roc), a kinase domain, and a WD40 domain, which contributes to the protein’s overall function. LRRK2 can bind and hydrolyze GTP, and is, therefore, an active GTPase capable of phosphorylating a wide range of substrates [17]. Phosphoproteomic studies have identified a subset of Rab GTPases, including Rab3A-D, Rab8A, Rab8B, Rab10, Rab12, Rab29, Rab35, and Rab43, as LRRK2 substrates [117]. Rab GTPases activated through phosphorylation by LRRK2 play a fundamental role in membrane and vesicle trafficking by regulating lysosomal homeostasis processes [118]. Therefore, LRRK2 is key in numerous signaling pathways involving vesicular trafficking, such as the endolysosomal and autophagic systems [119]. Although the exact physiological role of this protein is not yet fully understood, it has been shown that it is a cytosolic protein that is physiologically recruited to the membranes of stressed lysosomes where it facilitates interaction with effector proteins and maintains their homeostasis [120]. LRRK2 is a ubiquitous protein expressed in all tissues, with the highest expression in the brain, lung, and kidney. In particular, it has been suggested that its brain expression is relatively high in the substantia nigra and ventral tegmental area of the midbrain where dopaminergic neurons are abundant [121]. At the brain level, LRRK2 plays an important role in the control of synaptic vesicle trafficking [122]. Beyond its physiological role, it has been proposed that LRRK2 could contribute to the development and progression of various pathologies, particularly those related to neurodegenerative disorders [123,124]. Autosomal dominant missense mutations in the *LRRK2* gene are one of the most prevalent genetic causes of PD, and data from genome-wide association studies have identified common variants in the LRRK2 locus on chromosome 12 linked to increased risk of idiopathic disease [125]. These mutations are located within the enzymatic core of LRRK2 and the most common is G2019S, which causes a dysregulation of enzymatic activity translated into increased kinase activity [126]. Indeed, LRRK2 overexpression has been observed in numerous postmortem brain samples from patients with idiopathic PD. In PD patients, in addition to the G2019S mutation in the kinase domain, which shows increased kinase activity of LRRK2, there is also the R1441C mutation. The R1441C mutation in the GTPase domain could decrease the GTPase activity of LRRK2 [35,127]. However, conflicting evidence suggests that the LRRK2 R1441C mutation increases kinase activity [35,128]. Primary cortical neuronal cultures expressing the LRRK2 R1441C mutation have been shown to exhibit reduced autophagy-lysosomal fusion, altered lysosomal pH, and reduced lysosomal protein degradation, deficits not seen in wild-type LRRK2 cells [129]. Although LRRK2 hyperactivity is closely related to the development of PD, to date it is still unclear how these LRRK2 mutations lead to the neurodegeneration characteristic of PD. Recent studies in rats overexpressing LRRK2 have demonstrated that increased enzymatic activity leads to impaired autophagosome transport, indicating defects in the degradation of autophagosomal cargo in cortical neurons and the hippocampus [130,131]. This is supported by a different study that found that autophagosome transport was disrupted in LRRK2 knock-in mouse models [132]. Furthermore, LRRK2 mutations are closely associated with mitochondrial dysfunction linked to changes in the intracellular transport of mitochondria and impairment of the mitophagy pathway characteristic of PD pathogenesis [133]. Therefore, overexpression of LRRK2 impairs mitophagy, which may impair dopaminergic neurons, stimulate neuroinflammatory processes, and increase oxidative stress typical of PD [134]. Furthermore, the disruption of axonal autophagy can also delay the degradation of α-syn, resulting in accumulation in the form of Lewy bodies present in Parkinson’s disease [135]. Confirming this, previous studies have shown high levels of α-syn aggregating in neurons with LRRK2 G2019S mutations [136,137]. In contrast, the deletion of LRRK2 suppressed the aggregation and somatic accumulation of α-synuclein, delaying the progression of neuropathology developed in genetically modified mice [138]. Although future studies are needed to identify the mechanistic link between LRRK2 and α-synuclein, it has been identified that the Rab35 GTPase substrate of LRRK2 could mediate LRRK2-induced neurodegeneration [139]. Indeed, the phosphorylation of Rab35 by LRRK2 increased α-synuclein aggregation in SH-SY5Y cells by altering the endolysosomal degradation pathway and causing amplification of the aggregates [140]. Therefore, based on this evidence, mutations in the protein kinase LRRK2 contribute to the onset and progression of PD.

### Roles of LRRK2 on Other Neurodegenerative Diseases

Increased tau phosphorylation has been reported in multiple cell and animal models expressing pathogenic LRRK2 mutants [141]. Therefore, LRRK2 kinase hyperactivity plays a role in neurodegeneration by disturbing microtubule dynamics contributing to the progression of AD. Furthermore, the transmembrane protein APP is cleaved to form various fragments, including the intracellular domain (AICD). In mouse models and in an vitro study, it was demonstrated that AICD promotes LRRK2 expression and activates LRRK2-mediated neurotoxicity via transcriptional coactivator forkhead box O3 (FOXO3a) [142].

Moreover, a clinical study demonstrated that subjects with PD and neurodegeneration of the anterior horn of the spinal cord, reminiscent of ALS, had LRRK2 mutations [143]. Therefore, the association of LRRK2 mutations with an ALS-like phenotype inspired researchers to investigate whether LRRK2 variants are common in ALS patients. Whittle et al. demonstrated that in a cohort of ALS subjects, two common SNPs were also observed in the intronic region surrounding exon 35, although no evidence of splicing was observed at this position in LRRK2. Furthermore, no evidence of known pathogenic LRRK2 substitutions was observed in ALS patient samples. However, further genetic screening is necessary, as it cannot be excluded that other genetic variations in the LRRK2 locus may play a role in the pathogenesis of ALS [143].

LRRK2 has been shown to regulate voltage-gated calcium channels, responsible for the influx of Ca ions in response to membrane depolarization [144]. Since it is known that mitochondrial Ca dyshomeostasis is one of the main causes of striatal neurodegeneration in HD, a possible involvement of LRRK2 in this pathology has been hypothesized [145]. In the context of HD, autophagy plays a key role in lipid metabolism. Indeed, the inability to remove damaged or excess lipids through lipophagy could contribute to neurodegeneration [146]. Unfortunately, only one study that thoroughly examined this idea that showed postmortem human brain tissues with HD was found to contain some Lrrk2-positive intracytoplasmic and intranuclear inclusions [147]. Therefore, based on all this scientific evidence, the overexpression of LRRK2 in neuronal and glial inclusions in numerous neurodegenerative diseases suggests that these pathologies may share a common pathophysiological pathway.

## 5. Interplay Between CKs and LRRK2

**Figure 4:**
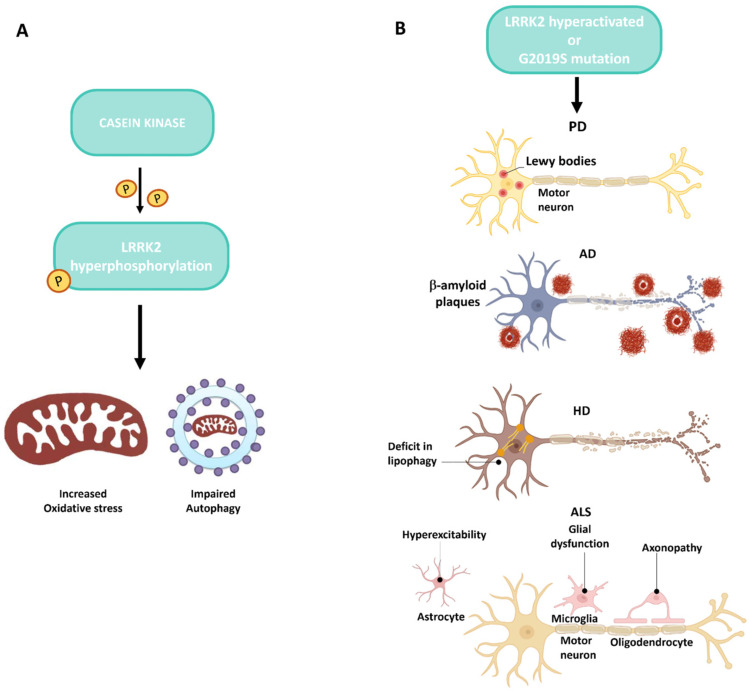
Interplay between CKs and LRRK2 in neurodegenerative diseases. (**A**) This panel represents how casein kinase increases LRRK2 phosphorylation. Hyperphosphorylation of the LRRK2 protein determines an increase in oxidative stress with a compromised autophagic process. (**B**) This panel represents the onset of neurodegenerative diseases following hyperactivation of LRRK2 and/or mutation of the *G2019S* gene.

In neurodegenerative diseases, studies targeting CK to regulate upstream LRRK2 activity could be of relevant interest given this close connection of these kinases both involved in CNS pathogenesis. Hyperactivation of LRRK2 can impair the autophagic process, contributing to the accumulation of toxic proteins and, consequently, to oxidative stress. These events are significantly involved in the pathogenesis of numerous neurodegenerative diseases (Figure 4).

However, it is not yet clear which upstream CK may regulate LRRK2. Although the physiological kinases and phosphatases acting on LRRK2 have to be fully characterized, recent scientific evidence has demonstrated the implication of CKs. Given the key roles of LRRK2 and CKs in the development of neurodegenerative diseases, clarifying their interaction link could be important for the search for new diagnostic and therapeutic markers of these pathologies. Phosphosite mapping studies via mass spectrometry support the idea that LRRK2 is a highly phosphorylated protein—additionally, these studies show at least 74 phosphorylation sites on the isolated LRRK2 protein, representing nearly 3% of all amino acid residues in the protein. The majority of LRRK2 phosphorylation sites include serine (59%), threonine (37%), and tyrosine (4%) residues, but half of these sites are still being confirmed. The balance of LRRK2 phosphorylation is also related to its cellular distribution [148,149]—therefore, elucidating the players involved in the regulation of LRRK2 phosphorylation is crucial to understanding how LRRK2 might be dysregulated and, consequently, how it influences signaling processes. It is now known that the main cellular partners of LRRK2 that regulate the phosphorylation state are protein kinases and phosphatases. The first kinase reported as a candidate to regulate LRRK2 phosphorylation was Protein-Kinase A (PKA) [18]. However, it was subsequently shown that the activation or inhibition of PKA had no effect on LRRK2 phosphorylation, and the recent literature supports the theory of functional cross-regulation between LRRK2 and PKA, which may be cell-type-specific. In the context of neuroinflammation, the kinase inhibitor IκB (IKKα and β) has been shown to phosphorylate S910 and S935 sites in bone-marrow-derived macrophages during the stimulation of toll-like receptor signaling. Furthermore, IKKβ is also a potential kinase that regulates LRRK2 phosphorylation in SH-SY5Y and HEK293 cells [17]. Recent data indicated that a kinase highly involved in the phosphorylation of LRRK2 at the brain level is CK1α. CK1α is a kinase regulator upstream of LRRK2 at constitutive phosphorylation sites. Identification of CK1α as a proximate kinase for LRRK2 at the neuronal level was performed by pharmacological inhibition of CK1 and siRNA for CK1 in HEK-293T cells and mouse brain with ex vivo experiment [18]. Initiation of CK1 resulted in dephosphorylation of LRRK2, while incubating purified LRRK2 with recombinant CK1α in kinase buffer in vitro showed that CK1α could phosphorylate LRRK2 not only at S910 and S935 but also at S955 and S973 [18]. Furthermore, treatment with siRNAs targeting CK1α also reduced the RAB29-dependent Golgi fragmentation caused by LRRK2. These data indicate that phosphorylation of LRRK2 sites by CK1 modulates the recruitment of LRRK2 within the Golgi [18]. Another study confirmed the interaction link between CK1 and LRRK2 in the neurological field. It was demonstrated that inhibiting the upstream kinase CK1α for 6 h in SHSY5Y cells expressing LRRK2 WT resulted in a significant induction of LRRK2 S935 dephosphorylation, accompanied by a strong reduction in total LRRK2 protein levels. In addition, LRRK2 kinase inhibition in SHSY5Y neuronal cells induced N-terminal changes and disrupted the LRRK2-CK1 interaction, leading to reduced heterologous phosphorylation and degradation of LRRK2 protein [150]. However, the effect of CK1 inhibition in vivo has achieved contradictory results. Indeed, CK1 inhibition in wild-type mice induced dephosphorylation of LRRK2 S935 in the lung and kidney but not in brain extracts. Nonetheless, it should also be considered that in the studies carried out so far, the CK1 inhibitors used are not specific for the CK1α isoform; therefore, we cannot exclude that other CK1 isoforms are involved in the regulation of the stability of the LRRK2 protein in different tissues. Another reason that should push research to investigate the crosstalk between LRRK2 and CKs in depth is linked to the fact that some variants of LRRK2 are insensitive to the destabilization induced by inhibitors of the LRRK2 kinase, while CK1 could act downstream of the inhibition of LRRK2 kinase, ultimately leading to the degradation of LRRK2, overcoming this obstacle [151]. In conclusion, LRRK2 kinase activity requires GTPase activity which functions independently of kinase activity, and it is known that both LRRK2 and GTPase kinase activity are required for neurotoxicity and potentiate neuronal death (Table 3).

## 6. Conclusions and Future Perspectives

In conclusion, LRRK2 kinase activity requires GTPase activity, which acts independently of kinase activity, and both LRRK2 and GTPase kinase activity are known to be required for neurotoxicity and enhanced neuronal death. In this review, we presented a novel entry point to understand the regulation of LRRK2 phosphorylation and the downstream functional links that correlate with neurodegeneration processes. Our overall interpretation is that kinase regulation of LRRK2 is a major event mediated by CK. Therefore, in neurodegenerative diseases, studies targeting CK to regulate the upstream activity of LRRK2 could be of relevant interest and need to be further discussed, given this close connection between both kinases involved in the pathogenesis and early onset of neurodegenerative diseases. However, due to the poor specificity of currently used inhibitors for LRRK2 and CK, there is a considerable lack of certainty in many experimental results. Indeed, since inhibitors could target other kinases or cellular proteins, the results may lead to ambiguous conclusions. Furthermore, CK enzymes possess a wide range of substrates, which makes it difficult to isolate and fully understand their specific roles in LRRK2 regulation. Finally, CK kinase expression is widespread in various tissues and cell types, which makes its functional analysis even more complex in the context of specific diseases such as Parkinson’s or other neurodegenerative pathologies. In light of these observations, it is evident that, although targeting CK to regulate the upstream activity of LRRK2 may represent a promising therapeutic strategy in neurodegenerative diseases, there is still much to explore and understand. Therefore, future studies that delve deeper into the interactions between CK and LRRK2 may be crucial to provide valuable insights for the development of new therapeutic strategies.

## Figures and Tables

**Table 1:** CK1 and CK2 involvement in PD.

Casein Kinase	Target Molecules	Activity	Consequences	Approaches
CK1	*Parkin* gene	Phosphorylation in Ser/Thr sites	Reduction of solubility and its aggregation	*CRISPR/Cas9 technique* **targets**: genomic loci **aim**: - understand the malfunction of genes and proteins involved in PD - provide the body with correct copies of the disease-causing gene
Dysregulation CK1		Amplify cellular stress and DNA damage	Increased mutations in *Parkin* gene	
CK2β	α-syn	Enhancing phosphorylation on Ser87 and Ser129	Co-localization in Lewy’s bodies	
CK1 and CK2	α-syn	Phosphorylation on Ser129	Accumulation Lewy’s bodies	

**Table 2:** Summary table of clinical and preclinical data on CK1 and CK2 in AD.

Casein Kinase	Alzheimer Study	Expression Levels	Activity	Consequences	Approaches
CK1α, CKδ and Cdε	Clinical study	Upregulation in hippocampal levels		Phosphorylation of tau protein and formation of neurofibrillary tangles	Genome editing studies to better understand how dysfunctional CK1 signaling is closely related to the development of AD
CK1ε isoform		Upregulation	Processing of the amyloid precursor protein (APP)	Accumulation of neuronal dopaminergic phosphoprotein (DARPP-32) phosphorylated by Thr-34	
CK2	Clinical study	Downregulation		Reduction of neuroprotective effect	
CK2	in vitro study	Upregulation	Directly phosphorylates Ser7 and Ser9 of presenilin-2 (PS-2)	Soluble APP	
CK2	in vivo study	Upregulation		Reduction of b-Amyloid plaques formation	
CK2 inhibitor (SET)	in vivo study	Downregulation	Inhibition of PP2A in cytoplasmatic level	Hyperphosphorylation of tau protein	
CK1δ and CK1ε inhibitor (PF670462)	in vivo study	Downregulation		Reduction of β-Amyloid plaques formation	

**Table 3:** Interconnection between CK1 activity and LRRK2 phosphorylation.

Molecules	Activity	Golgi Apparatus Level	Final Effect
GTPase	Activation	LRRK2	Neurotoxicity/Neuronal death
Activators/Inhibitors	Targeting PKA	LRRK2 S910/S935	No effect on LRRK2 phosphorylation
Initialization CK1α	Reduced LRRK2 degradation	Enhancing phosphorylation on Ser87 and Ser129	Co-localization in Lewy’s bodies
CK1 and CK2	LRRK2 S910/S935/S955/S973	Phosphorylation on Ser129	Accumulation Lewy’s bodies
siRNA	Targeting CK1α	LRRK2	reduced RAB29-dependent Golgi fragmentation caused LRRK2
